# Desensitization induces hyporesponsiveness and cell-surface phenotype changes on wild-type and humanized mouse mast cells

**DOI:** 10.1186/1939-4551-8-S1-A217

**Published:** 2015-04-08

**Authors:** Pedro Giavina-Bianchi, Matthieu Picard, Veronica Mezzano, Joana Caiado, David Edward Sloane, Mariana Castells

**Affiliations:** 1 Brigham and Women's Hospital, Harvard Medical School, USA

## Background

Rapid drug desensitization (DST) protocols have been developed based on clinical evidence, but in vitro studies are lacking. Understanding the mechanisms involved in the early stages of DST will allow improvements in patients' treatment. The aim of this study is to demonstrate and characterize the induction of hyporesponsiveness in murine mast cells by desensitization.

## Methods

We assessed the effect of rapid desensitization on murine bone marrow derived mast cells (BMMC). Experiments with three diferent types of murine BMMC were done: wild-type, LILRB4-/-, and transgenic expressing the human receptor FceRI. Wild-type and LILRB4-/- BMMC were sensitized with anti-DNP IgE and stimulated with DNP, while transgenic BMMC were sensitized with concentrated human serum of allergic subjects and stimulated with the respective allergen. BMMC were stimulated through desensitization or single activation, with optimal or suboptimal doses of antigen. Comparisions were made according to the percentage of b-Hexosaminidase (b-Hex) release by BMMC and the expression of LAMP-1 on the surface of these cells. Furthermore, expression of FceRI, PDL1 and LILRB4 on the surface of BMMC were also assessed.

## Results

DST inhibited the IgEmediated degranulation of BMMCs as desensitized cells released 43.3% less βHex and showed 73.2% lower LAMP1 surface expression compared to activated BMMCs. A group of desensitized and activated BMMCs were challenged again with an extra dose of 1 ng DNPHAS and additional release of βHex and LAMP1 expresssion were not different from the negative control, regardless whether BMMCs had been previously desensitized or activated. The hyporesponsiveness state of BMMCs induced by DST and activation was not due to mediator depletion as calcium ionophore induced marked release of βHex in these cells. Activated and desensitized BMMCs, respectively, expressed 40.3% and 15.7% less FcεRI, 45.9% and 13.1% less PDL1, and 23.5% and 11.3% less GP49 than negative controls. We observed that while the expression of PDL1 on the cell membrane decreased, its intracellular amount increased.

## Conclusions

Desensitization and activation induced hyporesponsiveness in WT and humanized BMMC, which can be assessed by LAMP1 expression and β-Hex secretion. Hyporesponsiveness is not due to depletion of mediators or mediated by soluble factors, and is associated, but not dependent on FceRI internalization.

